# Opening Address

**Published:** 1844-12

**Authors:** Jas. Taylor


### ARTICLE II.

*Opening Address.* By Jas. Taylor., M. D., D. D. S. Delivered before the Mississippi Valley Association of Dental Surgeons, on the 14th of August, 1844.

*Mr. President and Gentlemen*
*of the Mississippi Valley Association of Dental Surgeons:*

I rise not to deal in the antiquities of our profession. The history of dental science, as well as that of medicine, comes not to us unobscured by the superstition of the dark age which closed on the bright and glorious dawn of the christian religion. It is not only necessary, but altogether professional, to know the history of the science we may cultivate; yet this knowledge may be obtained, and often is, and not the science itself.

I may know that the Egyptians, even before the rise of the Roman empire, had a regular organized dental profession, sustained by government, and made hereditary; I may know that the luxurious Greek, the elegant Athenian, looked upon a beautiful set of teeth as ornaments indispensable to grace and beauty. This and much more may be known, and yet be of no practical benefit.

I know not but that he from whom we all claim descent, may have had to endure all the tortures and agonies of the most racking and distressing of all complaints, "the tooth-ache." I think it quite likely that Adam did require the soothing care and medical skill of mother Eve. I cannot say she did or did not extract any of his teeth. The removal of a tooth may have been the first surgical operation ever performed. We will not, however, quarrel with our brethren of the surgical profession about the antiquity of our respective avocations. We will not, at the present, lift the veil from the early history of medical and dental science. We presume, however, that "necessity" is as much the mother of either science as "of invention," for when disease first made its appearance, necessity called for a remedy, and thus, till the present day, we go on from one experiment and discovery to another, until animated, as well as inanimated nature has been brought under the analyzing touch of man. Man's necessity has led to this. The wants of our physical system keep constantly drawing on the intellectual for not only the means of subsistence, but also of preservation.

We know, however, from "Holy Writ," that the "eating of the forbidden fruit" brought disease and death on us all, since which time it has been the lot of all mankind to suffer, from not only diseases of the dental organs, but also *all* the organs of the body.

There is such an intimate connection between the various parts of the system, that we can hardly conceive it possible that any one organ can be seriously affected, and the others (at least some of them) not suffer. Trace, if you please, the fifth pair of nerves throughout all its ramifications, and you will find its branches distributed to the eye, the ear, the superior and inferior maxillary—the teeth and the tongue, all running back to one grand centre, the crura of the cerebellum—other nerves, distributed to other parts of the system, link in, as it were, with the branches of this nerve, holding in direct and sympathetic union the whole organization.

Touch an inflamed dental nerve, and like an electric shock it is felt throughout the whole. These small delicate fibres, in which is centred the sensibilities of the system—vibrate at every touch—are susceptible to every impression, yielding either pleasurable or painful sensations. How various the impressions received? How exquisite their sensibility—disturb them not, and the lulled storm lays not more tranquil on the "expanded ocean"—agitate or disturb their proper functions, and the raging storm shows not more signs of agony and commotion.

How important then that every organ perform its proper office—that none should be disturbed by the ravages of disease, but that health should reign in every part of the system. Then, and only then, can the equilibrium be sustained, and life's vigorous action preserved, without this, decay "creeps apace," energy after energy is destroyed, until disease completes its purpose and every organ falls its intended prey.

The teeth may properly be called the first organs in the digestive apparatus. If they fail to perform their office well, the balance are called on for an undue proportion of labor, this, in time, impairs their power, and thus dyspepsia, with a long train of nervous affections commence their work of destruction.

It is supposed that a healthy state of the saliva is necessary for the proper digestion of our food; with a diseased condition of the teeth and gums, what else must we look for but a vitiated condition of this fluid? How small soever the cause of disease, yet let it constantly operate, and it will ultimately produce its effects. "The constant dripping of water wears away in time the hardest rock." Let one faculty of the mind after another be impaired, and reason at length will be dethroned.

There is a point beyond which the strongest cord will not bear stretching, one thread after another yields, until it snaps asunder. Just so with the brittle thread of life, cord after cord yields to disease, until at last the king of terrors takes easy possession.

What constitutional derangement takes place in the formation of a small abscess? High arterial excitement, rigors, followed by fever and general prostration of the system. I have known a simple alveolar abscess produce such a high degree of fever, preceded by such violent chills, as to be taken by the attending physician, for a violent attack of intermittent fever.

How numerous are the cases of cancer, of sarcomatous and fungous tumors, produced by dental irritation? How frightful are sometimes the diseases of the antrum, often caused, too, by one diseased and useless tooth? Take a case where one of the bicuspides penetrates the antrum Highmorianum. This tooth becomes diseased, the nerve suppurates, disease seats itself at the point of the root on the periosteum, within the antrum. In this condition the tooth is filled. The matter constantly forming at the point of the fang is thus confined. A sack forms and bursts within the cavity. Here, from a slight operation, improperly performed, a troublesome and even dangerous disease is induced. It is true this might and often does take place without the interference of the dentist, yet, in such a condition, plugging would be very improper. I merely draw attention to this case to show the importance of a proper knowledge of the parts on which we operate.

When we take into consideration the delicate and complicated structure of the mouth—so many organs, so completely dependant on each other, if not for life at least, for healthy action—the periosteum enveloping, as it were, the whole dental structure—its immediate connection with the alveoli, the gums and the contiguous parts, including the parotid and maxillary glands, when thus viewed in connection with the amount of dental disease constantly occurring, we have cause to rejoice that so few fatal cases occur.

Think not that I magnify the importance of dental operations. For it is with the present state of dental science, and the importance of a thorough medical dental education I wish to deal. I would that I could present this subject in its true and practical bearing. I view it as all the other branches of medicine and operative surgery, incalculable in its benefits when properly understood and applied, often exceedingly injurious and destructive when practised by the ignorant and unskilful.

But, am I asked, what has all this to do with the present state of dental science? I answer, much.

For dentistry now teaches, that not only all the bad effects I have named, but many others, proceed from a diseased condition of the dental organs. Fitch enumerates the following, as being produced by this cause:

"Phthisis pulmonalis, dyspepsia, pain in the ear, and formation of matter in that organ, opthalmia, nervous affections, epilepsy, hysteria, hypochondriasis, rheumatic affections, tic douloureux and sympathetic head-ache." I stop not to inquire how all these diseases are thus produced. I have no doubt but that they are all, and many more, if not thus produced, at least aggravated thereby. Perhaps it would be far easier to trace the cause of any of the diseases named in the dental irritation, than to the influence of malaria, yet how often do we see, in a diseased condition of the mouth, all the elements operating necessary to the production of malaria. This, too, is a malaria constantly operating not only on the lungs, but is taken into the stomach in every act of deglutition. This, too, is a malaria which operates on the highest peaks of our mountains, as well as in the low and muddy swamps of the west, in all latitudes, in all locations, it is constantly exerting its baneful influence, requiring, in all conditions of life, care and cleanliness to avoid its production, and the consequent evils attendant thereon.

Thus we find in every case of scurvy, (so called,) two causes operating conjointly on the system to bring on some other form of disease. We have not only the irritation and debilitating effects of the disease itself, but the constant influence of a morbific fluid taken into the stomach, and the inhalation of the effluvia (or malaria) of this morbific matter into the lungs. Thus operating on the stomach and lungs at one and the same time. We hear of persons gradually sinking into the grave, from the effects of an abscess or of some other local disease. But how seldom is the loss of life attributed to the effects of alveolar abscess, where, perhaps, the amount of matter formed, and irritation produced, twice exceeds that in the former case. The mouth, indeed, by some, appears to be considered as a general receptacle for any and every thing, requiring not half the care and attention to keep it clean as the feet or hands.

Mr. President, I have thus alludud to the effects of dental disease the better to understand what is required of us as members of the dental profession. I hope it may enable us to form some idea of the importance of dental science. The duties of our profession certainly require that we should properly understand these things, and be prepared, pathologically, to meet the various diseases of the teeth, and their appendages, with suitable remedies.

This brings me more particularly to that part of my subject on which I intend calling the especial attention of this society, viz. a medico-dental education.

We see there is something more wanted than merely a knowledge of the manipulations we are called on to perform—something to direct us when, why and wherefore, we should operate. The organs which come under our care are parts of the organism which make up the whole of our physical system, subject alike to disease, and exerting, when diseased, an influence on other organs highly injurious and destructive to their healthy action. The conclusion, then, is inevitable, that a "thorough medico-dental education" is absolutely necessary to all who may engage in the practice of our profession.

What then constitutes a "medico-dental education?" It is that knowledge of medical science which shall enable us properly to understand the diseases of the dental organs—the effects of such disease on the general constitution—the causes, whether local or constitutional, which operate to produce disease, and the remedial agents necessary for the prevention of such diseases and their cure.

This might be called the medical department of our profession, and with this I shall first occupy the attention of this society, after which, make a few remarks in relation to mechanical dentistry.

The first thing, then, which claims our attention is the medical department of our profession. This is the foundation on which we must build our superstructure. *Here* commences the labors of the dental student—*here* his investigations should be thorough and complete—for much of his practice must be based on the principles here entertained; how important then that the first principles of any profession should be based on the sure foundation of truth.

The first thing presented to the dental student is a tooth. He wishes to know the constituent principles of its composition. On inspection, he finds it formed, apparently, of two substances, enamel and bone—but the question still comes up, of what are these composed? To determine this, a knowledge of chemistry must be obtained, and not only is such knowledge requisite to enable him to analyze the tooth, but also to determine what external agents may act injuriously on the enamel or bone. Without such knowledge, he knows not what remedies he dare use as a dentifrice, nor how to compound or prepare such as he may desire. There is not, indeed, a local remedy—lotion, powder, or any thing of the kind which he may find necessary to use—but requires a knowledge of chemistry to determine the manner of application or the practicability of its use.

Is the knowledge of chemistry important to the medical practitioner? equally, if not more so, is it for the dentist. Ignorance on this subject has ruined thousands of teeth.

The use of acids in tooth-powders and washes for the mouth, would never be used nor prescribed by one who properly understood this subject. Any acid, having a greater affinity for lime than the phosphoric, should never be used incautiously about the teeth. The natural tendency would be their decomposition.

The next thing which attracts the student's attention, is the organization of the teeth.

Are they supplied with nerves, blood vessels? Also, secreting and excreting vessels? How formed? How kept in their place? How connected with the surrounding parts? What parts are injured in their removal? How can hæmorrhage take place, and what is the peculiar process of dentition? Before all these questions are properly answered, he will find himself at least to the "*head and ears*" in anatomy, and that its study is indispensable before he can rightly understand this part of his profession. Methinks, if a surgeon was about to amputate an arm, he would first like to know what muscles were to be severed, what nerves to be cut—what arteries to be tied, and whether one or two bones were to be sawed. He would also like to know how the operation might affect the system, and what would be the consequences were he to enclose a nerve with the artery in his ligature.

He who would undertake such an operation, without the requisite knowledge, would be considered as the veriest empiric, a bold and daring quack, trifling with the life of a fellow-creature. Is not the mouth as important as an arm? If so, just as necessary then is it that the dentist should understand the anatomy of the parts on which he operates.

Let it not be thought, however, that it is only necessary to understand the anatomy of the dental organs, for other organs are intimately connected with them, and are liable to more or less injury by improper operations on the teeth—dental disease affects not only the teeth but sometimes the whole system. This cannot be properly understood without a knowledge of the system itself. He must understand the connection of various organs of the body—their relative bearing on each other—their nervous and vascular connection, before he can estimate the effect of a diseased action in one organ on another. Without this he cannot account for the various symptoms which may present themselves, nor determine what are the remedies indicated for cure. So then, as the wants of dental science are traced out, physiology and pathology come in for a share of our attention. But we stop not here. The diseases of the teeth require not only local but constitutional remedies. So varied are the derangements they produce, that to treat successfully the different cases that may be presented, a knowledge of practical medicine and therapeutical pharmacy will be required. Is the physician required to study pharmacy and the theory and practice of medicine, that he may designate disease, pronounce upon its prognosis and diagnosis, and be enabled to prepare and give his remedies? Why then should not the dentist be required to do the same thing? A man would hardly undertake to treat the diseases of the eye, without having first studied anatomy, the general principles of medicine, and whatever appertains to the general studies of the medical student. Let me ask, then, what does the "present state of dental science require?" It requires that the dental student should study chemistry, anatomy, pathology, physiology, the general principles of practical medicine and pharmacy. That he should have as thorough a knowledge of all these subjects as the practitioner of medicine, I do not pretend to say. But that he is competent to practice his profession without some considerable knowledge of all these branches of medical science, I utterly deny.

I know that many of those who have adorned our profession, and will ever be considered as bright luminaries of dental science, were not regular graduated doctors, yet that they were ever content to practice the profession as mere mechanical dentists, we know was not the case. Strange would it be that those who elevated dentistry from the condition of that, as an unimportant art, to the rank it now assumes as one of the most important and useful of the professions—strange, indeed, would it be that such men should have neglected the study of all science which would throw light on their favorite pursuit. The present condition of dental science, as well as some of their writings, plainly tell us *such was not the case.*

Such men as Koecker, Parmly, Waite, Hudson and our late lamented brother, professor Hayden of Baltimore, never could consent to neglect this part of their profession, and if I mistake not, some of these gentlemen, by their learning and general information on medical as well as dental science, have acquired the honorary titles of doctors of medicine.

If any man doubts the importance of a thorough anatomical and pathological knowledge of the head and neck, let him peruse a work by Mr. A. Burns, with additional cases and observations by professor Pattison of the University of Maryland, on tumors, &c. of the head and neck, and I think he will see ample cause for all the information he can acquire on that particular part of the system. How many cases there recorded, an intelligent dentist might trace to dental irritation, I cannot say, yet I think he will find several, where a proper and early attention to the teeth would have prevented the necessity of a tedious, painful and dangerous operation.

One case, in particular, where professor Mott, of New York, was under the necessity of removing one complete half of the inferior maxillary, in consequence of a disease of that bone and the contiguous parts. I am sorry that the early history of this case is not more fully given, yet there is enough to impress upon my mind the conviction, that the protrusion of the dens sapientiæ had much to do in promoting the disease, and its removal at the proper time, with, perhaps, the second molar, might have saved the young lady all the pain she endured, and the loss of half of the lower jaw. It is true that no dentist may have been called on to prevent any of the frightful cases given in the work alluded to—indeed, such is often the insidious approach of such diseases, that no medical assistance is *called* for until too firmly seated to be easily removed.

The success of the operation above alluded to, as well as others given, show the splendid achievements of some of the best surgeons of any age, let us hope that the *achievements* of dental science may be such as to prevent the necessity for many such operations for the future.

Professor Harris of Baltimore, will, I hope, soon give to the profession, the history of a case, where the removal of two teeth cured entirely, in a few weeks, a tumor of the superior maxillary, after it was thought that nothing but the removal of the larger portion of that bone could effect a cure.

In looking over the work already alluded to, I was much gratified to find, that our late professional brother, Dr. Hayden of Baltimore, was the first to trace out the true nature of a disease, an aneurism of the branches of the internal maxillary artery, and directed the patient to professor Pattison, who succeeded in preserving the life of a valuable young man, by tying the great carotid artery. This case had been treated, by the attending physicians, for another disease, and an operation to remove by cutting away the tumor, must, in all probability, have occasioned instant death. Dr. Hayden's accurate knowledge of the parts, enabled him to pronounce it an aneurism, while professor Pattison's experience *enabled him* to determine upon it as an anastomosing aneurism, which it proved to be.

It is with feelings of some professional pride, that I look back to the life and important services of this departed brother. Settled in his profession, at an early day, in the monumental city, where, indeed, but little was known about the importance of dental operations—by an honorable, conscientious and upright course, secured not only the respect and confidence of the public, as a man, but by the success of his operations, proved to the world that dental operations, when properly performed, are incalculable in their benefits.

He needs not an eulogium from me, for he lived to see his services appreciated, and while the noble monuments of human art were going up around him, failed not to establish for himself a monument of fame more enduring than those of marble, whose pointed spires adorn the city wherein his own was acquired. While we deplore his loss, let us, like him, strive to place our profession in that elevated position, in the confidence of the public, which its true merits so eminently deserve.

In the second place, it is necessary that the dental student should get a correct knowledge of mechanical dentistry. This, I must confess, is no easy matter—by some it can never be acquired. There is a mechanical tact, so called; a dexterous use of instruments; a correct judgment and a good eye, without which no man can succeed in dental operations. It not only requires close observation, and a correct knowledge of the manner in which every operation should be performed, but considerable use of the instruments to enable him to succeed so as to satisfy himself and his patient. Even after all this is acquired, he will continually go astray unless guided by sound principles on the theory of his profession. Also, of moral rectitude.

I would say, that two years is scarce sufficient time to acquire a good knowledge of medical and mechanical dentistry.

I believe that many of our best operators would concur with me in saying, that after two years! four or six years' patient investigation and laborious practice, much useful knowledge can be acquired.

How utterly ridiculous then the idea, that a tuition of a few months, or, perhaps, only a few weeks, is sufficient to enable any man, with success, to perform operations, which require years of close application and study. Dentistry, to be respected and confided in, can never be made what it should be, and must be, until this all-absorbing evil is removed. The members of the profession, those who have skill, and are capable of giving instruction, must unitedly set their faces against this short method of manufacturing "scientific dentists" out of any and every kind of *material*. Then, and not till then, can we expect an elevated standard of professional attainment.

The whole of this vast continent (save I believe two states) is open to the depredations of these mushroom sprouts of dental science.

A confiding public demand of us some protection against their repeated impositions, the honor of our profession, and suffering humanity require that we should do something to eradicate this evil. Let then the probation be such, as shall ensure hereafter requisite qualifications. Demand of every student such a course of study as shall make him understand the profession he intends to practice.

Perhaps there is no subject connected with dentistry which the student, at first, so little appreciates, as that of the mechanical department of the profession. It is true, that many think that this alone is necessary, and this they expect to acquire in two or three weeks! How often do we have such applications, young men who wish to study dentistry in a *few weeks?* Yes, we frequently see natural doctors and dentists—prodigies in the medical and dental professions—"natural geniuses," whose natal star rises and sets in the magic circle of seven—in whose healing touch recuperation unfolds the vigor of youth and beauty—disease quails before their approach, and the king of terrors, in affright, abandons his intended victim. These gentry, thus endowed, scarcely think it necessary to plod through the regular routine of scientific investigation, but at once step from the anvil, the bench, or the plough, to the highest ladder of professional attainments—fame's loudest trumpet proclaims their skill—every breeze wafts abroad some dreadful case relieved—the public press groans beneath the mighty burden of their infallibility, and the world is astonished to see the "mountain in labor bring forth a mouse." What care they, if the "trumpet of fame" is blown by their own mighty lungs? Or the public press weighed down by the fevered hallucinations of their disordered brain—their object is pelf, and with this they expect to gild their conscience, and buy the reputation of public benefactors. Many, from their insignificance or contempt, are permitted to pursue their course through "life's boisterous journey," and none to say "what doest thou?" There is a vice which suffers not approach, a corruption which bears not handling, and a species of quackery which cries health! health! when death's awful visage is scarcely hid beneath the specious folds of his pompous charlatanism.

Shall our favorite science any longer be disgraced by his obsequious bearing? If we dare not tear away his robes and expose his hideous deformity, let us, at least, not suffer him to enter our dwelling and pollute the temple of our science. Let the contempt of an honorable profession rest upon him, until he die neglected, scorned and degraded, an object of loathing and disgust to all lovers of science. Let *us*, too, remember, that much of the quackery which disgrace the practice of dentistry can be traced to the low estimation which members of the profession generally place upon the necessary qualifications, requisite for its practice. What estimation can be placed on a profession by one who will undertake to teach it in a few weeks or months? He must either expect his student to be extra smart, or *feel* that *he* has very little to impart. The speed of the steam car or magnetic telegraph cannot be applied to the acquisition of science.

Dental science, in the last few years, has been disrobed of many of the absurdities which clung to her for centuries. The superstition of the "dark age" had enveloped in mystery almost every principle of the healing art.

The conjuror, with a few herbs and incantations, for a long time reigned over medical science, and dentistry sank beneath the grip of the blacksmith and the "barber's basin."

The incantations of the conjuror shrank from the light of scientific research. The iron grip of the blacksmith, in mercy, relaxed at the havoc he had made, and the barber's basin, thanks to Don Quixotte, and his faithful Sancho, found better employ in the days of old Spain's knight errantry. Dentistry, thus released, rose from her bed of sorrow, shook off the dust and rubbish of her long degraded bondage, and now stands forth attired in the rich drapery of science. Hunter threw at her feet a mind stored with years of knowledge. Bell threw around her a rich garment culled from the store-room of pathology. Koecker, Fitch, and a host of other worthy names have drawn from truth's great laboratory, some choice gems to adorn her brow; shall we not assist to weave a rich and splendid robe for her attire, and twine a wreath to deck the diadem she wears?

Mr. President, and gentlemen of the Mississippi Valley Association of dental surgeons, we have embarked in a praiseworthy undertaking. Let us, with unanimity, carry out the design of our meeting. It is no less than the elevation of dental science, and the protection of the public from imposition.

No selfish feeling prompted us to this undertaking—let no such feeling retard the good work we have commenced.

Let us look straight-forward, having in view the honor of our profession, and the amelioration of the pains and afflictions of our fellow-men, and, ultimately, we shall receive their warmest commendation.

We have already entered on the arduous duties and responsibilities which devolve upon us as practitioners of an important branch of medical science. The medical faculty look on our enterprise with favor, ever ready and willing to lend a helping-hand, when the honor of their profession and the public good demand it. Let us then do *our duty* in perfecting this science, until the skill of man shall stop only in obedience to Him who sends afflictions on us for our good, and has thrown around us His rich bounties for our relief in sickness, and support in health.

The fair temple of dental science has opened wide her doors, and is ready to receive at her shrine all those who offer true love and affection. She accepts no divided love, but demands of all her votaries assiduous care and attention.

We have laid on her altar our youthful desires and aspirations. Have we not been warmed in the sun-shine of her favor, and cheered by the benefits she conferred.

She has yet veiled vast stores of hidden treasure. Let us lift this veil, and cull her choicest fruits. At every turn rich gems of knowledge beckon us onward. Let us grasp these jewels. Let us press onward, explore well the magnificent temple, bring to light all the "hidden treasure," and leave for after-ages this temple, in all its apartments, too beautiful to be touched by the rude hands of quackery, ignorance and superstition.

